# The effect of some missing teeth on a subjects' oral health related quality of life

**DOI:** 10.12669/pjms.346.15706

**Published:** 2018

**Authors:** Shafi Ullah Khan, Fazal Ghani, Zulfiqar Nazir

**Affiliations:** 1 *Shafi Ullah Khan, BDS, MCPS, FCPS. Department of Prosthodontics, Khyber College of Dentistry, University Campus, Peshawar, Pakistan*; 2 *Fazal Ghani, Ph.D., FDSRCPSGlasg, M.Sc, CMP, BDS, BSc. Professor, Head of Department of Prosthodontics & Dean Postgraduate Dental Sciences, Peshawar Dental College, Peshawar - Pakistan*; 3 *Zulfiqar Nazir, BDS, FCPS Resident. Department of Prosthodontics, Bacha Khan Dental College, Mardan - Pakistan*

**Keywords:** Oral health related quality of life (OHRQoL), General Oral Health Assessment Index (GOHAI), Missing teeth, Partial edentulism

## Abstract

**Objectives:**

To determine the impact of missing teeth on the level of Oral Health Related Quality of Life (OHRQoL)in subjects reporting at a teaching dental hospital.

**Method:**

Using a structured Performa incorporating the 12-item General Oral Health Assessment Index (GOHAI) Questionnaire, and a consecutive (non-probability) sampling technique, data relating to 182 subjects fulfilling the study inclusion and exclusion criteria were collected using the method of interview and examination. Subjects responses to each of the 12 items of the GOHAI questionnaire were recorded to determine the impact of missing teeth on OHRQoL. Each of the GOHAI item had a maximum score of 5 thus giving a total of 60 as the maximum score. A high score of GOHAI indicated better ORHRQoL. The ORHRQol of subject was taken as good when the GOHAI score ranged 57-60, average when 51-56 and poor when ≤50.

**Results:**

Subjects had a mean age of 35.6 ± 5.8 (S. Dev) with males as 50.5% compared to females (49.5%). The mean GOHAI score for all the subjects was 48.4 ± 8.2 as compared to the mean GOHAI score of 48.4 ± 8.2 for males and 47.6 ± 8.3 for females. The ORHRQoL was good in only 27%. A high proportion of subjects (53%) had poor OHRQoL. The number and the frontal location of the missing teeth adversely impacted OHRQoL. Missing maxillary anterior teeth had the most negative effect on OHRQoL. Missing mandibular first molar was the most common missing tooth either alone or in combination with other missing teeth among the subjects studied. The most important GOHAI items contributing to the adverse impact on the OHRQoL of the majority of subjects with some missing teeth were;*often worried/concerned about dental problems* and never having been *pleased or happy with the look of their teeth and gum*

**Conclusion:**

The adverse effect of missing teeth on OHRQoL was substantial necessitating the importance of preventing the condition of missing teeth or restoring when missing and maintaining the oral health of subjects.

## INTRODUCTION

Health is no longer seen solely as the absence of illness and disease. Rather it is the complete physiological, psychological and social well-being of a person.^1^ Health related quality of life is an emerging subject of importance during recent years.^2^ This is based on realization that the effects of a disease or condition cannot be fully determined by using solely clinical measures; since these do not take in to consideration the subjective experiences that individuals have concerning their health.^2^

Oral health is an integral part of the overall health of individuals. Oral health related quality of life (OHRQoL) can be defined as the part of quality of life that is affected by person’s oral health.^1^,^2^ OHRQoL captures how oral health affects person’s ability to function (e.g. chewing, speaking), his psychosocial status and the related pain and discomfort. It is increasingly accepted that measurement of OHRQoL is an essential component of oral health surveys, clinical trials and other studies evaluating the outcome of preventive and therapeutic programs intended to improve oral health.^2^ Indeed it is becoming accepted that problems in oral health can create significant complications and cost not only for the specific individual but also for the healthcare provider and the society as a whole. Thus it is for the overall good of both the individual and society that quality of life is taken into account. Oral health status can be affected by many personal, social and local factors.

Differences in oral health status can be seen when comparing different region within the country or between countries and geographical location. OHRQoL is adversely affected by tooth loss, untreated dental decay, periodontal disease and other pathologies and abnormalities within stomatognathic system.^3^ The loss of teeth and poor dentition is known to affect the mastication of foods and nutritional status. The loss of teeth is known to adversely affect occlusal forces; chewing ability especially in subject not wearing dental prostheses.^4^ Facial attractiveness has been found to affect attitudes and action and is important in employment situations. Missing and decayed teeth drastically affect the appearance of person. This results in negative impression on prospective employers and poor self-esteem for individuals. Additionally, adverse oral health conditions have been found to affect systemic health, quality of life and economic productivity.^4^

A local survey showed that an increase in number of missing teeth, low socio-economic status and low education level had negative impact on OHRQoL.^4^ Another local study showed that 32.2% patients seeking prosthodontics treatment had problem in mastication.^5^ It has been shown that fewer than nine teeth had more impact on OHRQoL than having cancer, hypertension and allergy.^6^ It has also been shown that the effect of factors on OHRQoL of a group of subjects was seen as difficulty in chewing in 35.1%, difficulty in showing teeth while smiling in 17.5% and difficulty in enjoying the social contact with people in 18.2%.^1^

OHRQoL is assessed by using questionnaires including the Oral Health Impact Profile (OHIP), the Oral Impact on Daily Performance (OIDP) and the well-established original Geriatric Oral Health Assessment Index (GOHAI) now called as General Oral Health Assessment Index (GOHAI.^2^,^4^,^7^,^8^

The GOHAI is a 12-item assessment questionnaire originally developed by Atchinson and Dolan in 1990 in USA for use with elderly population. It was later renamed the General Oral Health Assessment Index by Atchinson in 1997so as to enable its use in all adults.^2^,^7^,^8^ The 12-items of GOHAI are grouped into the following three fields including;

Functional field (eating, speaking and swallowing), psychological field (concern about oral health, dissatisfaction with appearance, self-conscious about oral health and avoidance of social contacts because of oral problem) and pain or discomfort field (drugs, gingival sensitivity, teeth sensitivity, discomfort when chewing certain foods).^2^,^7^,^8^ GOHAI has been translated and validated in various languages including Persian, Chinese, Arabic, German, and Malay.^1^,^3^,^8^-^10^

Despite GOHAI having been used so widely for the assessment of ORHRQoL in many countries, there is little work done locally about the negative impact of missing teeth on the GOHAI Score and hence ORHRQoL in local populace. Therefore, this study aims to investigate the effect missing teeth might have on the GOHAI score and hence ORHRQoL of a sample of subjects reporting to the teaching clinic at the Prosthodontics Department, Khyber College of Dentistry, Peshawar (Pakistan).

## METHODS

Approval of hospital’s ethics committee was taken. Subjects referred from the outpatients department to the prosthodontic clinic and fulfilling the inclusion and exclusion criteria with some missing teeth in one or both jaws were invited to participate in the study. The purpose, risks and benefits of study was explained to each subject and informed consent was taken regarding their willingness and participation in the study. Strict exclusion criteria were followed to control confounders and bias in the study. The socio-demographic as well as data for the number and location of missing teeth was recorded. The modified form of the original English version of the GOHAI questionnaire was translated into local language (Pashto) by senior faculty at the Pashto Academy University of Peshawar. The GOHAI questionnaire was completed for each subject by one investigator (SK) through the method of interviewing and clinical examination. The responses from the participants was recorded on a 5-point Likert (1= always, 2=often, 3=sometimes, 4=seldom; 5=never). Responses to statements items 3, 5 and 7 were reverse scored. The method used in this study was cumulative method (GOHAI-Add), which consist of summing up the scores obtained for each of the 12 GOHAI questions / statement. Using these cumulative (GOHAI-Add) score, ORHRQoL of a subject was determined as good if the was 7-60), average when 51-56 and poor when ≤50.

Data were collected and analyzed by SPSS version 17. Descriptive statistics were done to compute the mean and standard deviation for age and GOHAI score. Frequencies and percentages were calculated for gender, grading on GOHAI scoring index (good, average, poor). OHRQoL based on GOHAI index was stratified among age, gender, number of missing teeth and location of missing teeth to see the effect modifications. All results were presented in the form of tables and graphs.

## RESULTS

Among the 182 subjects, 111(61%) were in age range 30-35 years, 29 (16%) in age range 36-40 years and 42 (23%) in age range 41-45 years. Mean age was 35.6± 5.8 years. Males were 92 (50.5%) and females were 90 (49.5%) giving a male to female ratio of almost 1:1.

**Fig.1:**
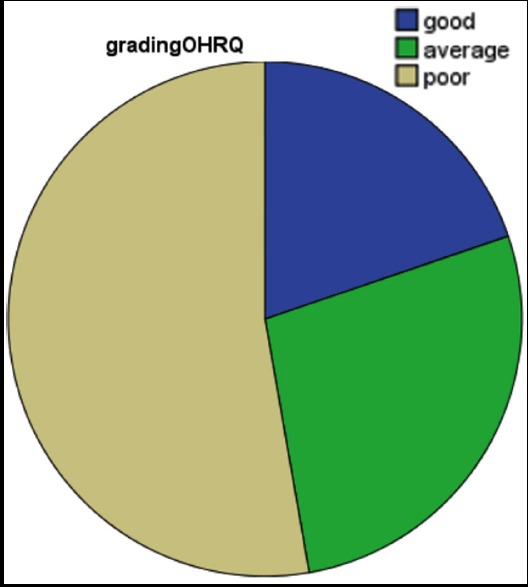
Levels of oral health related quality of life (OHRQoL) of the subject with missing teeth (N= 182).

The GOHAI score ranged as 22-60 with a mean score of 48 ± 8.3. Thirty six (20%) subjects had good OHRQoL(GOHAI score 57-60), 50 (27%) subjects had average OHRQoL (GOHAI score 51-56) while 96 (53%) had poor OHRQoL (22-50)

When GOHAI grading and score was stratified with age, no significant correlation was found between OHRQoL and age. There was only a weak relation between OHRQoL and gender. The mean GOHAI score for males was 48.43 ± 8.2, and for female was 47.6 ± 8.4showing missing teeth had more negative impact on OHRQoL in females.

The more the number missing teeth in subject, the lower was the GOHAI score and hence a relatively poor OHRQoL. The detail description regarding correlation between GOHAI grading and score and the number of missing teeth are given inTable-I. Regarding the impact of the location of the missing teeth, maxillary anterior missing teeth had a more negative effect on OHRQoL than the missing teeth in the other location of the jaws.

**Table-I:** Relationship of the numbers and GOHAI Score.

* NTM	No. of Subjects	Mean GOHAI Score
1	46	52.3±6.8
2	34	51.9±6.7
3	18	47.8±5.9
4	17	48.1±6.6
5	15	46.1±8.7
6	7	45.6±8.3
7	6	46.2±5.7
8	7	46.0±7.9
9	2	45.0±8.5
10	7	42.1±8.4
11	2	40.0±12.7
12	5	35.2±5.6
13	2	45.5±10.6
14	4	44.5±5.1
15	2	23.0±1.4
16	1	46.0±0.0
18	2	39.0±7.7
19	2	39.0±1.4
20	1	39.0±0.0
23	1	43.0±0.0
26	1	36,0±0.0
Total	182	48.0±8.3

* NMT= number of missing teeth, Chi square test p-value 0.001.

The distributions of responses to the GOHAI items are presented in Table-II. These shows that irrespective of the levels of problems, the most important GOHAI item which impacted the OHRQoL of a great majority of partially dentate subjects were problems related to; esthetic, chewing, concern about the missing teeth. Item that can be regarded least important in terms of impacting the subjects’ ORHRQoL were; swallowing, speaking and being conscious/ nervous. Other items that had a moderate impact on OHRQoL included; limiting amount of food and worried about missing teeth.

**Table-II:** No. of subjects (%) and their GOHAI scores for each GOHAI item.

S. No	GOHAI Items	No. of patients (%) in each score level				
		Never 5	Seldom 4	Sometimes 3	Often 2	Always 1
1	How often did you limit the kinds or amounts of food you eat because of problems with your teeth?	103 (56.6)	34 (18.2)	14 (7.7)	12 (6.6)	19 (10.4)
2	How often did you have difficulty biting or chewing any kinds of food, such as firm meat or apples?	62 (34)	59 (29)	16 (8.8)	23 (12.7)	28 (15.5)
3	How often were you able to swallow comfortably?	3 (1.6)	3 (1.6)	2 (1)	8 (4.4)	166 (91.4)
4	How often have your teeth prevented you from speaking the way you wanted?	126 (69.2)	36 (19.4)	4 (2.4)	7 (4)	9 (5)
5	How often were you able to eat anything without feeling discomfort?	6 (3.1)	16 (8.8)	27 (14.8)	54 (29.7)	79 (43.6)
6	How often did you limit contacts with people because of the condition of your teeth?	127 (69.8)	19 (10.4)	5 (2.7)	14 (7.7)	17 (9.4)
7	How often were you pleased or happy with the look of your teeth and gums?	37 (20.3)	29 (16)	13 (7.1)	36 (19.8)	67 (36.8)
8	How often did you use medication to relieve pain or discomfort from around your mouth?	79 (43.4)	92 (50.5)	6 (3.3)	4 (2.2)	1 (0.6)
9	How often were you worried or concerned about the problems with your teeth, gums?	17 (9.3)	53 (29.1)	24 (13.2)	22 (12.1)	66 (36.3)
10	How often did you feel nervous or self-conscious because of problems with your teeth, gums?	117 (64.3)	42 (23)	4 (2.2)	9 (5)	10 (5.5)
11	How often did you feel uncomfortable eating in front of people because of problems with your teeth?	121 (66.5)	35 (19.2)	7 (3.8)	10 (5.5)	9 (5)
12	How often were your teeth or gums sensitive to hot, cold or sweet foods?	87 (47.8)	63 (34.6)	10 (5.5)	14 (7.7)	8 (4.4)

## DISCUSSION

It was noted that Oral health related quality of life (OHRQoL) is adversely affected by the condition of partial edentulism with an associated of the number, location and distribution of missing teeth. Age has been considered an important variable affecting OHRQoL so this could have acted as a confounder in this study.^3^,^4^,^8^ For this reason all the subjects selected for this study were in age range of 30-45 years which is considered as an age group when a person is young, free of diseases, have chosen a carrier and is independent.

It was noted that 20% subjects had good OHRQoL, 27% patients average OHRQoL while 53% patients had poor OHRQoL. Similar results were found in other studies. In one study on 160 subjects in Saudi Arabia (age 60-90 years), mean GOHAI score was 32.1 (range 11-59) with the mean number of 14 missing teeth per patient (1-29). Among the subjects 21.8% were having good/satisfactory, 40.1% were having average and 37.8% were having poor OHRQoL.^8^ The mean GOHAI score in this study was 48. In one of the study in North Jordan in which 288 participants completed the Arabic version of GOHAI, the mean GOHAI score was 40.9 and their mean age was 33.4±13.2 with some 62% of them had at least one missing tooth.^9^ The difference in GOHAI score which was higher in our study can be explained by age, cultural differences and mean number of missing teeth per subject. The age range of subjects included in this study was 35-45 years which is relatively younger group than that of the other two studies in Saudi Arabia^8^ and North Jordan.^9^

When the effect of age was compared with ORHRQoL, the results showed that this did not have a significant effect on the subjects OHRQoL. These results corroborate the findings of a local study done by Ghani and Khan in Pakistan (2010) and Inukai et al in Tokyo (2010) where OHRQoL was not substantially influenced by age.^4^, ^11^ But the other studies results showed a substantial effect of the age on OHRQoL.^3^,^4^,^6^ This difference can be due to the reason that the age range selected for this study was 30 to 45 years which is considered as the adult age and people are usually energetic, independent and free of diseases. With the passage of time the aging leads to more oral problems.

In this study when the gender was compared with the GOHAI score there was slight association between the GOHAI score and the gender. TheGOHAI score for the males was 48.4 and for the females it was 47.6 which indicate a relatively more pronounced impact of the missing teeth in the females. These findings are in accordance with the other previous local study by Ghani and Khan,^4^ Carneiro,^12^ and Ingle (India).^13^

The mandibular first molars were the most frequently missing teeth followed by the mandibular second molar. The number of missing teeth was found to be a strong factor for impaired OHRQoL as measured by GOHAI score. These results are supported by studies conducted by Ghani and Khan^4^, Gerritsen,^6^ and Ingle et al.^13^

Our results about the correlation of the missing teeth and the OHRQoL suggest that all of the lost teeth might not be equally important in their influence on OHRQoL. The reason for this is the stronger aesthetic importance of teeth in the anterior region especially in the maxillary anterior region which is one of the most important domains in patients need and wishes.^6^ Out of the 27 patients who had missing anterior, 21 rated their OHRQol poor, one subject labeled it good and five labeled it average. Similar findings were reported by Craddock (2009).^14^ The patient who had one missing anterior tooth reported low scores for GOHAI items compared to patients who had two or three missing teeth in the posterior region. This may reveal that an anterior missing tooth makes a subject so conscious about aesthetic that the concern of posterior missing teeth is reduced. Similar results were also reported from adults in a local study by Ghani and Khan,^4^ Craddock^14^ and Sheiham.^15^ The findings of this study indicate that GOHAI scoring of subjects fulfills the expectations of an instrument for a structured approach for identification of patient demands and needs and is thus an important screening tool in the decision making and treatment planning for restoring missing teeth.

### Limitations of the study

While the results of this study indicate the usefulness of the GOHAI scoring of patients as an important screening tool in the decision making and treatment planning for restoring missing teeth, these finding come from a small size single center populace and hence for these findings to be generalizable it would require the conduction of a larger study with the inclusion of a large populace from multiple centers and areas.

## CONCLUSIONS

Within the limitations of this study, the following can be concluded;

1. GOHAI scoring of patients may be an important screening tool in decision making and treatment planning for restoring missing teeth.
2. GOHAI scores influence OHRQoL of a subject. ORHRQoL is substantially affected by missing teeth and is significantly affected as the number of missing teeth increases and when the missing teeth are located in the frontal part of the maxillary arch.
3. Age and gender of the subject was not significantly factor for OHRQoL.

### Authors’ Contribution

All the authors **(SUK, FG & NZ**) actively contributed to the concept, design, study conduct and data analysis & writing and editing of the manuscript.

**SUK and ZN** were actively involved in data collection and initial draft writing of the manuscript.

**FG,** did data analyses and data interpretation and final editing, reviewing and approval of the man-uscript and has the responsibility to be the corresponding author.
